# Narrow-Band Imaging for the Detection of Oral Potentially Malignant Disorders and Early-Stage Oral Squamous Cell Carcinoma

**DOI:** 10.3390/jcm15093382

**Published:** 2026-04-28

**Authors:** Agata Świątek, Adrian Maj, Aida Kusiak

**Affiliations:** Department of Periodontology, Medical University of Gdańsk, 80-211 Gdańsk, Poland

**Keywords:** narrow-band imaging, oral mucosa, oral potentially malignant disorders, oral cancer, vascular patterns

## Abstract

**Background**: Early detection of oral potentially malignant disorders (OPMDs) and early-stage oral squamous cell carcinoma (OSCC) remains a major clinical challenge, as initial lesions often present with subtle or nonspecific findings during conventional white-light examination. Narrow-band imaging (NBI) enhances visualization of mucosal microvasculature and may improve the identification of dysplastic and malignant transformation. **Methods**: A narrative review of the literature was conducted in the PubMed, Scopus and Google Scholar databases. Studies published between January 2012 and January 2025 evaluating clinical applications of NBI in oral mucosal lesions, OPMDs, or OSCC were included. **Results**: NBI enhances visualization of intraepithelial papillary capillary loops (IPCLs), whose morphological alterations correlate with epithelial dysplasia and malignant transformation. Evidence suggests high diagnostic sensitivity (up to 87–100%) and specificity (approximately 83–96%) for detecting high-grade dysplasia and early OSCC. NBI also improves biopsy site selection, reduces sampling error, and supports surveillance of high-risk patients. **Conclusions**: NBI represents a valuable adjunctive diagnostic tool in oral medicine and dentistry. Although it does not replace histopathological examination, its integration into clinical assessment may enhance early cancer detection and improve management of patients with OPMDs.

## 1. Introduction

Oral cancer remains a major clinical and epidemiological challenge worldwide, characterized by high incidence and an unfavorable prognosis, particularly in cases diagnosed at advanced stages of disease. Despite advances in the diagnosis and treatment of head and neck malignancies, the five-year survival rate for patients with oral cancers remains low, largely due to delayed diagnosis and nonspecific clinical appearance of lesions in the early stages of disease.

Oral potentially malignant disorders (e.g., leukoplakia, erythroplakia, and oral lichen planus) may remain asymptomatic and present with subtle features that are difficult to interpret under white-light examination. Early detection and accurate assessment of these lesions are crucial for preventing malignant transformation and improving patient prognosis. Consequently, there is ongoing research for diagnostic methods that may assist clinicians in identifying high-risk lesions and in precisely selecting sites for biopsy.

Narrow-band imaging is a non-invasive endoscopic technique used in medicine to help clinicians diagnose cancerous lesions of mucosal surfaces. It is used in various medical fields, for example in gastroenterology, laryngology, urology, pneumology and other medical fields.

Narrow-band imaging uses the emission of specific narrow light bands, mostly blue or green, enabling detailed visualization of the vascular architecture of the oral mucosa. By selectively enhancing mucosal microvasculature, NBI allows the identification of abnormal vascular patterns that may reflect dysplastic or malignant changes in oral mucosa and early stages of OSCC. NBI represents a valuable adjunct to conventional clinical examination and a tool for guiding diagnostic and therapeutic decision making.

The aim of this article is to review the current literature on the application of narrow-band imaging in the diagnosis of oral mucosa lesions, with particular emphasis on potentially malignant disorders and early-stage OSCC, as well as to discuss the advantages, limitations and potential clinical applications of this technique in routine dental and periodontal practice. This review aims not only to summarize current evidence but also to critically evaluate the clinical applicability and limitations of NBI in oral diagnostics.

## 2. Materials and Methods

A narrative review of the literature was conducted using the PubMed, Scopus and Google Scholar databases. Articles published between January 2012 and January 2025 were considered in order to capture both the initial development and the most recent clinical applications of narrow-band imaging in the oral cavity. This study is a narrative review. Elements of the PRISMA guidelines were applied to enhance transparency and structure of the literature search and study selection process.

The search strategy included combinations of the following keywords: “narrow band imaging”, “oral mucosa”, “oral potentially malignant disorders”, “oral cancer”, and “vascular patterns”. Only articles published in English were included.

Studies were selected based on their relevance to the clinical application of narrow-band imaging in the assessment of oral mucosal lesions, OPMDs and OSCC. Non-clinical studies, case reports, conference abstracts, letters to the editor and studies not related to the oral cavity were excluded.

## 3. Results

### 3.1. Optical Principles of Narrow-Band Imaging

**Figure 1:**
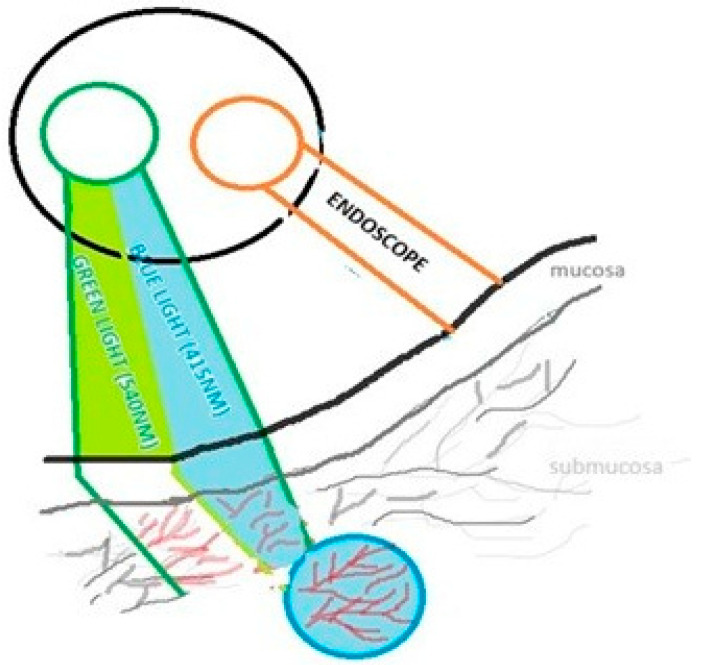
Principle of narrow-band imaging schematically showing selective visualization of superficial and submucosal vessels due to hemoglobin absorption of specific wavelengths. Different colors are used to distinguish conventional white light from the narrow-band blue (≈415 nm) and green (≈540 nm) wavelengths, highlighting their selective role in enhancing vascular visualization in NBI compared to standard imaging. Created by Authors.

Narrow-band imaging (NBI) is an endoscopic technique that enhances the visualization of mucosal microvasculature by using light at specific narrow wavelengths, typically blue light (415 nm) and green light (540 nm). These wavelengths correspond to the absorption of hemoglobin, which functions as a natural chromophore. Hemoglobin absorbs the light; thus, images of superficial and submucosal blood vessels obtained using NBI show increased contrast. Blue light (415 nm) penetrates only superficially, highlighting capillaries in the mucosal layer, while green light (540 nm) penetrates deeper, visualizing submucosal vessels. Vessels and epithelium are more visible on the background of surrounding tissues as it is shown in the Figure 1. It enables the detection of subtle architectural changes and abnormal vascular patterns that may indicate dysplasia or early malignancy not visible under white-light examination [1].

This technique is well tolerated; however, optimal image quality requires perpendicular illumination within 3 mm of the mucosa, without saliva or blood.

NBI images may reveal vascular patterns indicative of dysplasia or neoplasia. Abnormal mucosa shows well-defined brownish vascular patterns along capillary loops. In neoplastic lesions, the number, size, and intensity of brownish capillaries increase. Therefore, a specific classification based on the morphology of lesions observed in NBI has been developed. The key indicator of potentially malignant and malignant lesions in the oral mucosa is intrapapillary capillary loops (IPCLs) [2].

### 3.2. Intraepithelial Papillary Capillary Loop (IPCL) Classification Systems

Several classification systems of NBI images have been proposed to help clinicians provide accurate diagnosis of the disease, but currently there is no consensus for the classification criteria. NBI is used in the diagnosis and classification of lesions located in the area of head and neck, including oral, pharyngeal, laryngeal and sinonasal diseases.

**Figure 2:**
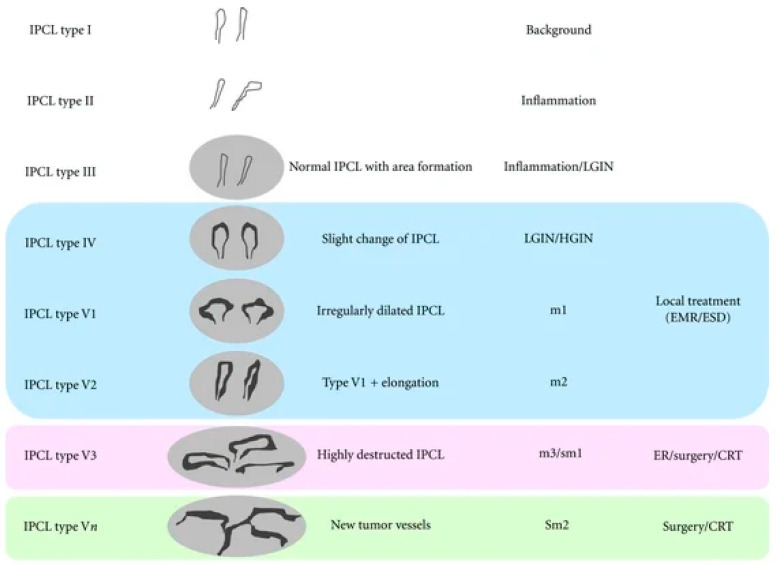
Intrapapillary capillary loop (IPCL) classification in narrow-band imaging (NBI) and its correlation with histopathology, invasion depth, and treatment strategy by Inoue classification (2001). Types I–III correspond to normal or inflammatory mucosa. Type IV is associated with low-grade and high-grade intraepithelial neoplasia (LGIN/HGIN). Type V reflects neoplastic changes and is subdivided into V1–Vn according to increasing vascular irregularity and invasion depth: V1 and V2 correspond to superficial mucosal invasion (m1–m2) and are typically managed with endoscopic treatment (EMR/ESD); V3 indicates deeper invasion (m3/sm1), requiring endoscopic resection or surgery/chemoradiotherapy (CRT); and Vn represents newly formed tumor vessels associated with deep submucosal invasion (sm2), usually requiring surgery or CRT.

This article focuses on the classification of oral lesions; therefore, the IPCL classification of the oral mucosa, as summarized by Takano based on Inoue’s classification, mention in the Figure 2, is presented below. The morphology of the IPCL is divided into four types (I to IV) [3]. The key vascular patterns observed in narrow-band imaging (NBI) and their diagnostic relevance are summarized in Table 1. In general, normal mucosa is characterized by regular, thin capillary networks, whereas dysplastic and neoplastic lesions demonstrate progressively disorganized and irregular vascular patterns.

Based on the classification originally proposed by Inoue and adapted to oral mucosa by Takano et al., IPCL morphology is divided into four types (I–IV) [3]:

- Type I—Regular, thin, loop-shaped capillaries with uniform caliber and distribution, corresponding to normal oral mucosa.
- Type II—Dilated and slightly elongated IPCLs, often located at a greater distance from the lesion margin; typically associated with reactive or low-grade epithelial changes.
- Type III—Markedly elongated, dilated, and tortuous IPCLs forming irregular or tangled patterns; frequently correlated with moderate epithelial dysplasia.
- Type IV—Severely distorted, destructured, or absent loop formations replaced by irregular neoplastic vasculature; strongly associated with high-grade dysplasia and invasive squamous cell carcinoma.

Progression from type I to type IV reflects increasing architectural disorganization, neoangiogenesis, and disruption of normal microvascular loops due to tumor invasion. This classification has been described as a clinically useful tool and non-invasive method for early detection and risk stratification of OPMDs.

Type I appears as a waved line with both waved arms and histologically occurs in the normal mucosa. Type II vessels are dilated, exhibit larger dimensions, and are located father from the lesion than those observed in Type I. Type III has elongated IPCLs that are accompanied by dilation. They can occur as long or tangled lines because of an increase in length. Type IV reflects the progression of carcinogenesis, with terminal branch loops becoming dilated, elongated, and ultimately disrupted in the final stage [3]. 

Previous studies focused only on the histological aspects; therefore, Tirelli suggested that it is important to consider the various epithelial structures [4]. Because of epithelial structures in the different oral sites and the different tissues he proposed six vascular patterns on NBI.

Intrapapillary capillary loops (IPCLs) represent the key microvascular structures evaluated in narrow-band imaging (NBI). Their morphology reflects progressive epithelial and subepithelial alterations associated with dysplasia and malignant transformation.

In 2012 Lin et al. made a classification of the epithelium, which explains that different sites of the same organ have different images on NBI. There were four types of epithelia pointed in this classification—type 1 keratinized thick stratified squamous, type 2a nonkeratinized thin stratified squamous; type 2b nonkeratinized very thick stratified squamous; and type 3 pseudo-stratified ciliated columnar epithelium [5].

### 3.3. Diagnostic Accuracy and Predictive Value of NBI

In NBI, normal oral mucosa demonstrates regular superficial capillaries and submucosal veins. IPCLs are single loops originating from an arborescent vascular network. When dysplasia occurs the structure of the vessels changes dynamically because of metabolic exchanges. The IPCLs initially elongate and dilate and when the tumor invades the submucosa, they are destroyed and replaced by irregular neo-tumor vasculature [3]. These vascular variations correlate with the degree of epithelial dysplasia and the progression of OSCC, providing non-invasive methods for early detection and risk stratification.

Several studies have demonstrated the clinical relevance of vascular patterns. Alterations in IPCL morphology differentiate benign from malignant changes in oral mucosal lesions. More complicated and complex IPCL patterns are strongly associated with dysplasia and early squamous cell carcinoma [6]. Meta-analyses indicate that IPCL grade II or above is a positive indicator that NBI achieves high diagnostic sensitivity (approximately 87%) and specificity (approximately 83%) for malignant transformation identification in OPMDs [7]. Abnormal vascular patterns in erythroplakia are correlated with higher grades of dysplasia confirmed by histopathology [8].

A prospective study by Deganello et al. described diagnostic accuracy of narrow-band imaging on patients with Oral Lichen Planus [9] focused on the identification of high-grade dysplasia in newly developed lesions. Newly developed lesions resistant to medical treatment were examined with the NBI light. All patients in this study underwent biopsy, which was defined as ‘positive’ in cases with high-grade dysplasia or carcinoma. The diagnostic potential of NBI appears to be high, with sensitivity of 100%, specificity of 96%, positive predictive in 71% and negative predictive in 100%.

In another article written by Cozzani et al. [10] the conclusion was that NBI may increase the accuracy of detection of subclinical malignant transformation in OLP and encourage clinicians to perform biopsy in selected areas.

According to Guida et al. [11] NBI could help in the follow-up of patients with multiple lesions of OLP through detection of capillary pattern IV, which seems to be the most significantly associated with neoplastic epithelium.

Piazza et al. in the article from October 2016 [12] demonstrated that NBI as an ‘optical biopsy’ significantly reduces the rates of false positives and false negatives in diagnosis of oral cavity cancer compared with conventional oral examination and white light.

### 3.4. Application of NBI in OPMDs

OPMDs represent the primary clinical field in which NBI is considered particularly useful for early cancer detection. OPMDs such as leukoplakia, erythroplakia, and oral lichen planus are characterized by heterogeneous clinical presentation and unpredictable malignant transformation risk.

NBI allows visualization of subtle vascular abnormalities that frequently precede visible morphological change. Studies included in this review indicate that lesions demonstrating irregular, dilated, or destructured IPCL patterns are significantly more likely to harbor moderate-to-severe epithelial dysplasia.

Particularly promising results have been reported in patients with oral lichen planus. NBI examination enables identification of newly developed or treatment-resistant areas exhibiting suspicious vascular patterns, facilitating early biopsy and detection of high-grade dysplasia or carcinoma in situ. Several studies suggest that NBI-assisted surveillance may improve long-term monitoring of patients with chronic inflammatory mucosal diseases, in whom early neoplastic changes may otherwise remain clinically occult.

NBI enhances detection rates of OPMDs and early malignant lesions that could be missed during white light examination. In a systematic review by Kim et al. [13] it was described that NBI more precisely identifies dysplastic and malignant changes compared to white light examination, with higher sensitivity and specificity. According to Ota et al. [14] OLP and oral leukoplakia were observed in larger areas with NBI than with white-light examination.

Additionally, NBI frequently reveals lesion extension beyond clinically visible margins under white light, supporting more accurate delineation of biopsy sites and improving histopathological diagnostic yield.

### 3.5. Role of NBI in Early Detection of OSCC

Early-stage OSCC often develops from pre-existing OPMDs and may initially present without ulceration, induration, or obvious exophytic growth. Consequently, reliance solely on conventional visual inspection may contribute to delayed diagnosis.

NBI enhances detection of early OSCC by highlighting disorganized microvascular architecture associated with tumor-induced angiogenesis. Advanced IPCL patterns, including tortuous, irregular, or destructured vessels, strongly correlate with invasive carcinoma [1,9,15].

Clinical studies demonstrate that NBI functions as a form of “optical biopsy,” enabling clinicians to identify the most biologically active regions within lesions prior to tissue sampling. This approach improves accuracy of surgical planning, facilitates margin assessment, and may contribute to earlier therapeutic intervention [13].

Recent prospective evidence also suggests that NBI may improve the assessment of surgical margins in oral squamous cell carcinoma, potentially enhancing intraoperative decision-making and reducing the risk of residual disease. Sittitrai et al. in 2025 showed a significantly higher rate of clear superficial margin in patients in the NBI group (96.2%) than in the White-light group (80.8%) [16].

From a clinical perspective, integration of NBI into routine oral examination may be particularly beneficial in high-risk populations, including patients with prior head and neck cancer, tobacco or alcohol exposure, and long-standing mucosal lesions requiring periodic surveillance.

### 3.6. Comparison of NBI with Other Diagnostic Methods

Conventional oral examination under white light remains the primary screening method in dental and periodontal practice; however, its diagnostic accuracy is limited in the early stages of dysplasia and OSCC, when clinical changes are subtle or nonspecific. Early microvascular alterations associated with malignant transformation are frequently not visible under white light, which may result in delayed diagnosis or imprecise biopsy site selection [9,17].

Narrow-band imaging (NBI) enhances visualization of mucosal microvasculature by highlighting pathological vascular patterns, particularly alterations in intraepithelial papillary capillary loops (IPCLs). Multiple studies and meta-analyses have demonstrated that NBI shows higher sensitivity and specificity for detecting dysplastic and malignant changes in OPMDs compared with white-light examination alone [14,17]. Additionally, NBI enables real-time guidance of targeted biopsies, improving diagnostic yield and reducing sampling errors [3,9].

Toluidine blue staining has long been used as an adjunctive screening method due to its ability to highlight areas of increased cellular activity. Although inexpensive and easy to apply, this method is associated with a relatively high false-positive rate, particularly in inflammatory or traumatic lesions [1]. In contrast, NBI provides functional vascular information rather than surface staining, which may offer improved lesion characterization and specificity.

Autofluorescence-based imaging systems detect metabolic and structural changes in oral tissues but are limited by low specificity, as fluorescence loss may also occur in benign inflammatory or hyperkeratotic conditions [5]. Compared with autofluorescence, NBI demonstrates superior correlation with histopathological findings, as microvascular abnormalities visualized by NBI are more closely linked to dysplasia and malignant transformation [9,14].

Despite its advantages, NBI does not replace histopathological examination, which remains the gold standard for definitive diagnosis [8,13]. Instead, NBI should be regarded as a complementary diagnostic tool that enhances conventional examination, optimizes biopsy site selection, and supports early detection and surveillance of high-risk patients [8,14]. The key characteristics, advantages, and limitations of NBI in comparison with other diagnostic methods for oral mucosal lesions are summarized in Table 2.

### 3.7. Clinical Implications for Dental and Periodontal Practice

Early detection remains the most important determinant of prognosis in OSCC. In this context, NBI is a valuable optical technique used adjunctively in the clinical assessment of high-risk mucosal lesions. It visualizes mucosal and submucosal vasculature which is not fully detectable with conventional white light examination. This enhanced imaging has led to better detection, diagnosis and management of OPMDs and OSCC.

One of the most clinically relevant applications of NBI is the improvement of biopsy site selection. OPMDs often present as large, heterogeneous lesions in which dysplastic transformation may occur focally. Conventional white-light examination may fail to identify the most representative area for biopsy, increasing the risk of sampling error. By highlighting abnormal microvascular patterns, particularly advanced IPCL alterations, NBI allows clinicians to identify areas with the highest probability of dysplasia or early malignant transformation [1].

NBI may also play an important role in the surveillance of high-risk patient populations. Individuals with long-standing OPMDs, previous head and neck malignancies, or chronic inflammatory mucosal diseases such as oral lichen planus require regular monitoring due to increased risk of malignant transformation. The ability of NBI to detect subtle vascular abnormalities before overt clinical changes become visible may facilitate earlier intervention and improved clinical outcomes [18].

From a practical perspective, NBI may be particularly useful in several clinical scenarios: evaluation of suspicious leukoplakic lesions, assessment of erythroplakia, monitoring of therapy-resistant oral lichen planus, and follow-up of patients treated for OSCC. In these situations, NBI can support clinical decision making and guide targeted biopsy, ultimately improving diagnostic accuracy.

However, appropriate interpretation of vascular patterns requires adequate training and experience. Therefore, the integration of NBI into routine dental and periodontal practice should be accompanied by standardized training and the development of clear diagnostic criteria.

### 3.8. Advantages and Limitations of NBI

NBI offers several clinical advantages as a non-invasive adjunctive technique for the evaluation of oral mucosal lesions. However it also has limitations that should be considered in the clinical use.

Advantages

(1) Enhanced visualization of vascular pattern.
(2) Improved diagnostic accuracy.
(3) Reduction in false positives and false negatives in lesion detection.
(4) Potential for early detection and surveillance.
(5) Guiding clinical decision making.

Limitations

Despite its advantages, NBI is not without limitations. The technique is highly operator-dependent and requires experience and training to accurately interpret vascular patterns [2,5]. Inflammatory conditions, ulceration, or trauma may result in false-positive findings due to reactive vascular changes [17,19]. Additionally, anatomical constraints of the oral cavity, presence of saliva or bleeding, and limited penetration depth may affect image quality [1,20]. Another significant limitation is the lack of a universally accepted and standardized NBI classification system for oral lesions, which complicates comparison between studies and limits widespread clinical adoption [2,13].

Moreover, heterogeneity among studies, including differences in study design, patient populations, and diagnostic criteria, as well as potential sources of bias, may influence the reported diagnostic performance of NBI [12,15]. These limitations should be carefully considered when interpreting the clinical applicability of NBI.

## 4. Discussion

Early detection of OPMDs and OSCC remains a major challenge in dental and periodontal practice, as early lesions often lack distinct clinical features under white-light examination [3,6]. Importantly, this review differs from previous publications by providing a focused synthesis of NBI applications specifically in oral mucosal lesions, with particular emphasis on clinical applicability in dental and periodontal practice. In addition, greater attention is given to diagnostic limitations, including the risk of overinterpretation in inflammatory conditions such as oral lichen planus, as well as the lack of standardized classification systems. This targeted approach aims to provide a more clinically relevant perspective compared to earlier reviews.

The diagnostic value of NBI is primarily related to its ability to reveal alterations in intraepithelial papillary capillary loops (IPCLs), which reflect underlying neoangiogenesis associated with epithelial dysplasia and malignant transformation. Evidence suggests that irregular, dilated, tortuous or destructured IPCL patterns are strongly correlated with high-grade dysplasia and early OSCC [3,6,10]. NBI has been reported to demonstrate high sensitivity and specificity for identifying malignant transformation in OPMDs, particularly when IPCL grade II or higher is considered a positive diagnostic criterion. These findings confirm that microvascular changes often precede overt morphological alterations detectable under white light examination [9,10,11,12,15].

The reported diagnostic performance should be interpreted with caution due to heterogeneity among studies, including differences in patient populations, lesion types and diagnostic thresholds [12,15]. In particular, differences in IPCL classification systems and variability in clinician experience may significantly influence reported diagnostic performance. Moreover, many studies are based on relatively small sample sizes or are conducted in specialized centers, which may limit the generalizability of the results to routine clinical practice.

An important clinical implication of NBI is its role in guiding targeted biopsy. Accurate biopsy site selection is critical, particularly in large or heterogeneous lesions. Evidence suggests that NBI-assisted biopsies improve diagnostic yield and reduce sampling errors by directing tissue collection to areas exhibiting the most suspicious vascular patterns [11,13,20]. This approach may decrease the number of unnecessary biopsies while increasing the likelihood of detecting high-grade dysplasia or early carcinoma.

The application of NBI in the follow-up and surveillance of high-risk patients appears promising. However, its role in chronic inflammatory conditions such as oral lichen planus (OLP) requires cautious interpretation [11,17,19,21]. Although OLP is classified as a potentially malignant disorder, the reported rate of malignant transformation remains relatively low, and the clinical behavior of these lesions is heterogeneous.

An important diagnostic challenge lies in distinguishing true OLP from oral lichenoid lesions (OLL), which may present with similar clinical and vascular features but differ in etiology and malignant potential. This distinction is not always clearly addressed in the available studies, potentially affecting the interpretation of NBI findings. Inflammatory changes in OLP may mimic dysplastic vascular patterns, increasing the risk of false-positive interpretation [11,17,19,21]. This may lead to overdiagnosis and unnecessary biopsies, particularly in cases where NBI findings are not correlated with clinical judgment and histopathological evaluation.

Therefore, while NBI may support the monitoring of selected high-risk lesions, its use in OLP should be considered as adjunctive and interpreted with caution within the broader clinical context [11,17,19,21].

One of the major limitations affecting the clinical implementation of narrow-band imaging (NBI) in oral diagnostics is the lack of a universally accepted classification system for vascular patterns. Several classification approaches have been proposed, including those adapted from gastrointestinal endoscopy (e.g., Inoue/Takano classification), as well as systems specifically developed for head and neck or oral mucosal lesions (e.g., Tirelli and Lin classifications).

The Inoue/Takano classification focuses on the morphology and arrangement of intraepithelial papillary capillary loops (IPCLs), categorizing vascular patterns according to progressive architectural disorganization associated with dysplasia and malignancy. In contrast, the Tirelli classification simplifies the assessment by grouping vascular patterns into clinically relevant categories, facilitating easier application in routine practice. The Lin classification further adapts these criteria to oral lesions, emphasizing practical differentiation between benign, dysplastic, and malignant changes. A comparative overview of these classification systems, including their main features, advantages, and limitations, is presented in Table 3. 

Notably, NBI should be regarded as an adjunctive diagnostic method rather than a replacement for histopathological examination. However, when integrated into routine clinical assessment, NBI has the potential to enhance early detection, improve risk stratification, and support more precise clinical decision making.

Future research should focus on the development of standardized oral NBI classification criteria and well-designed prospective studies. The integration of artificial intelligence with NBI may further improve diagnostic accuracy through automated analysis of vascular patterns. However, its clinical applicability requires validation in large clinical studies [2,5,22].

Overall, NBI has the potential to enhance early detection and clinical decision making in oral diagnostics, provided that its limitations are acknowledged and standardized approaches are implemented.

## 5. Conclusions

Narrow-band imaging is a valuable adjunctive tool for the evaluation of oral mucosal lesions, enhancing visualization of microvascular patterns associated with epithelial dysplasia and early OSCC.

It improves lesion detection and supports targeted biopsy and margin delineation. However, NBI does not replace histopathological confirmation and should be integrated within a multimodal diagnostic approach.

Further well-designed prospective studies are required to standardize diagnostic criteria and define its role in routine clinical practice.

## Figures and Tables

**Table 1:** Summary of vascular patterns in narrow-band imaging and their diagnostic relevance.

Pattern Category	NBI Vascular Features	Submucosal Veins	Corresponding Epithelium
Normal Type 1	Thin, branching dark green/brown capillaries; “flower-like” vascular endings	Occasionally visible	Keratinized thick stratified squamous
Normal Type 2a	Thin capillaries with acute branching angles	Clearly visible	Non-keratinized thin stratified squamous
Normal Type 2b	Regular punctate or short linear capillaries arranged equidistantly	Partially visible	Non-keratinized thick stratified squamous
Dysplastic (Type 1–2a)	Well-demarcated brownish/purple areas with thick dark spots	Absent within lesion; dilated vessels reaching lesion perpendicularly	Type 1 and 2a
Dysplastic (Type 2b)	Honeycomb or mesh-like vascular pattern	Absent within lesion	Type 2b
Neoplastic	Dark green spots, dilated winding or “bobby-pin” vessels; disorganized vasculature	Not distinguishable	Variable

**Table 2:** Comparison of NBI with other diagnostic methods for oral mucosal lesions.

Method	Principle	Advantages	Limitations	Clinical Role
White-light examination	Visual inspection of color, surface, and morphology	Widely available, non-invasive, first-line screening	Low sensitivity for early dysplasia, subjective	Baseline clinical assessment
Narrow-band imaging (NBI)	Visualization of mucosal microvasculature (IPCL patterns)	High sensitivity and specificity, real-time imaging, targeted biopsy guidance	Operator-dependent, limited availability, lack of standardization	Adjunctive diagnostic and surveillance tool
Toluidine blue staining	Staining of areas with increased nucleic acid content	Low cost, easy to use	High false-positive rate, influenced by inflammation	Preliminary screening aid
Autofluorescence imaging	Detection of tissue fluorescence changes	Non-invasive, wide-field assessment	Low specificity, affected by inflammation and keratinization	Adjunctive screening tool
Histopathological examination	Microscopic tissue analysis	Definitive diagnosis	Invasive, sampling error possible	Gold standard

**Table 3:** Overview and comparison of selected NBI classification systems for oral mucosal lesions, highlighting their principal characteristics, clinical advantages and limitations.

Classification	Main Feature	Advantage	Limitation
Inoue/Takano	detailed IPCL morphology	high sensitivity	complex, operator-dependent
Tirelli	simplified vascular patterns	easy to use clinically	less detailed
Lin	adapted for oral lesions	clinically relevant	limited validation

## Data Availability

No new data were created or analyzed in this study. Data sharing is not applicable in this study.
